# Acidification effects on biofouling communities: winners and losers

**DOI:** 10.1111/gcb.12841

**Published:** 2015-01-28

**Authors:** Lloyd S. Peck, Melody S. Clark, Deborah Power, João Reis, Frederico M. Batista, Elizabeth M. Harper

**Affiliations:** ^1^ British Antarctic SurveyHigh Cross, Madingley RdCambridgeCB3 0ETUK; ^2^ University of the AlgarveCtr Ciencias MarP‐8000139FaroPortugal; ^3^ Instituto Português do Mar e da Atmosfera (IPMA)Estação Experimental de Moluscicultura de TaviraVale Caranguejo8800TaviraPortugal; ^4^ Department of Earth SciencesUniversity of CambridgeDowning StreetCambridgeCB2 3EQUK

**Keywords:** algae, ascidian, assemblage, calcium carbonate, climate change, encrusting, ocean acidification, polychaete, skeleton, spirorbid, sponge

## Abstract

How ocean acidification affects marine life is a major concern for science and society. However, its impacts on encrusting biofouling communities, that are both the initial colonizers of hard substrata and of great economic importance, are almost unknown. We showed that community composition changed significantly, from 92% spirorbids, 3% ascidians and 4% sponges initially to 47% spirorbids, 23% ascidians and 29% sponges after 100 days in acidified conditions (pH 7.7). In low pH, numbers of the spirorbid *Neodexiospira pseudocorrugata* were reduced ×5 compared to controls. The two ascidians present behaved differently with *Aplidium* sp. decreasing ×10 in pH 7.7, whereas *Molgula* sp. numbers were ×4 higher in low pH than controls. Calcareous sponge (*Leucosolenia* sp.) numbers increased ×2.5 in pH 7.7 over controls. The diatom and filamentous algal community was also more poorly developed in the low pH treatments compared to controls. Colonization of new surfaces likewise showed large decreases in spirorbid numbers, but numbers of sponges and *Molgula* sp. increased. Spirorbid losses appeared due to both recruitment failure and loss of existing tubes. Spirorbid tubes are comprised of a loose prismatic fabric of calcite crystals. Loss of tube materials appeared due to changes in the binding matrix and not crystal dissolution, as SEM analyses showed crystal surfaces were not pitted or dissolved in low pH conditions. Biofouling communities face dramatic future changes with reductions in groups with hard exposed exoskeletons and domination by soft‐bodied ascidians and sponges.

## Introduction

Ocean acidification poses great potential threats to organisms and ecosystems (Doney *et al*., 2009; Constable *et al*., 2014). Negative impacts of acidified environments have been documented in several groups (Orr *et al*., 2005; Dupont *et al*., 2008; Byrne, 2011), with species whose calcium carbonate (CaCO_3_) skeletons are large proportions of their total biomass expected to be more strongly affected (Royal Society, 2005), especially in early developmental stages (Dupont *et al*., 2008); but data supporting either contention are equivocal (Ries *et al*., 2009; Kroeker *et al*., 2010). One problem is that much of our knowledge is based on single species studies, which may be useful for identifying underlying mechanisms, but tell us little about the effects of lowered seawater pH on communities (Hale *et al*., 2011).

Evaluations of impacts on communities and the identification of susceptible assemblages are crucial to predicting responses of and impacts on ecosystems. To date, such assessments are rare and outcomes usually unclear (Dijkstra *et al*., 2011; Hale *et al*., 2011). Encrusting biofouling communities are ideal test systems, as they include species with CaCO_3_ exoskeletons through to those lacking hard structures. This community is important worldwide, being the main colonizers and transformers of new surfaces in shallow marine environments. They also have great economic importance. In 2008 alone, the costs of managing and preventing marine biofouling were estimated at $15 billion for desalination systems and power plants and $7 billion for shipping worldwide (Jackson, 2008). The major biofouling organisms are sessile encrusting groups, typically bryozoans, calcareous tube‐dwelling polychaetes, sponges, ascidians and hydrozoans. Within these groups, several taxa, including spirorbid polychaetes, celleporellid bryozoans and sea squirts of the genus *Ascidia*, are unusual in having extremely large or global ranges. Understanding how this community responds to altered environments, especially acidified conditions, is thus important both scientifically and economically.

There is a repeatable succession in the development of biofouling communities on new surfaces (Watson & Barnes, 2004). Calcareous polychaetes are often amongst the most prominent early metazoan colonizers (Stark, 2008). However, the factors governing succession are complex, and the effects of changed conditions remain unknown. Here, we aimed to investigate the effects of lowered pH (7.7), compared to controls (ambient, pH 7.9), on both established community structure and on the development of communities on newly exposed surfaces in a flow‐through, pH controlled experimental aquarium system. We used the biofouling community from the Ria Formosa Lagoon in southern Portugal as our test system. We further aimed to investigate these effects over an extended 100‐day period that covered multiple generations of the commonest species.

## Materials and methods

Experiments were performed in the experimental station of the Centre of Marine Science in the Ria Formosa lagoon, Portugal. The facilities are licensed for animal experimentation, and the experiments were covered by a Group‐1 licence (Direcção‐Geral de Veterinária, Portugal).

### Culture system

The flow‐through holding system consisted of six independently supplied and operated tanks all at 23 °C, with 3 at control pH (7.9) and 3 low pH (7.7), that is three independent replicates, for each pH treatment. Sea water was supplied from the Ria Formosa lagoon via a sand filter that removed all particles larger than 1.2 mm diameter, and performed partial removal down to 0.6 mm. This size range is significantly larger than the minimum dimensions of most polychaete and ascidian larvae (Stanwell‐Smith *et al*., 1997). Each experimental tank was aerated and received 150 cm^3^ min^−1^ of seawater from the header tank, maintained at 23 °C using an aquarium heater (NEWATT; Aquarium systems, Sarrebourg, France) equipped with a thermostat (±0.1 °C). Excess water overflowed, and the water in each tank was totally exchanged 3–4 times per day. Experimental and header tank temperatures were logged every 30 min (±0.1 °C, probe = ACQ210N‐TL; Aquatronica, Reggio Emilia, Italy). Seawater pH was continuously logged (ACQ210N‐PH; Aquatronica), and pH in experimental tanks was automatically controlled by CO_2_ injection into the tank aeration supply. Injected CO_2_ was controlled by an Aqua Medic pH Computer Set and solenoid valve. Experimental tanks were illuminated with daylight fluorescent lamps with a 12‐/12‐h light/dark regime. Conditions in the experimental system were stabilized for 1 month prior to initiation of experimental trials. CO_2_ and temperature were monitored and controlled in real time. Salinity was measured with a VWR EC300 conductivity meter (Carnaxide, Portugal), and pH was also measured daily with an OxyGuard Handy pH meter (Farum, Denmark).

The experimental circuit was fed with the microalgae *Isochrysis galbana* (clone T‐ISO, at 18 000 cells cm^−3^ per experiment), supplied in continuous flow to each tank by a peristaltic pump (ISMATEC, Wertheim, Germany). Chlorophyll‐a concentration was measured in each tank *in vivo*, using a portable fluorometer (10AU‐Turner Designs, Sunnyvale, CA, USA).

### Colonization evaluation and statistical analyses

Precolonized HDPE pipe and new surfaces of HDPE pipe, glass fibre tank walls and limestone tiles were all numbered to facilitate matching during the experiment. Photographs were taken of all substrata at the start and end of trials using a NIKON D80 (Tokyo, Japan) with NIKON DX SWMED IF Aspherical AF‐S NIKKOR 18–70 mm 1 : 3 5.5–4.5 GED lens. In precolonized trials, pipes were photographed and three sections analysed per tank (nine sections per pH treatment). For each section, spirorbids were counted in 8.25 cm^2^ areas (*n* = 10) and other taxa in 25 cm^2^ areas because of the difference in density between taxa. Values were then recalculated and expressed as numbers 10 cm^−2^. In all trials, there were zero values in some counts made, and data were not normally distributed even after log, double log or arcsin transformation. Data were therefore analysed using nonparametric Kruskal–Wallis tests with Bonferroni *P* value corrections when multiple tests were run.

### Sea water parameters

The following were measured: temperature (°C), salinity (ppm), plus total phosphate (μmol kg^−1^ seawater), total silicates (μmol kg^−1^ seawater), total alkalinity (TA: μmol kg^−1^ seawater), total carbon dioxide (DIC: μmol kg^−1^ seawater) (Table 1). Seawater quality was assessed weekly using commercial Aquarium test kits. Using this system, ammonia, nitrite and nitrates were maintained well below 0.4, 0.2 and 5 mg L^−1^, respectively.

**Table 1:** Mean (± SE) seawater treatment parameters for control and low pH trials

Seawater parameter	Control	Low pH
pH_NIST_	7.91 ± 0.03	7.70 ± 0.03
pH_TOTAL_	7.78 ± 0.03	7.57 ± 0.03
pH_SEAWATER_	7.77 ± 0.03	7.56 ± 0.03
Ω calcite	3.18 ± 0.16	2.10 ± 0.15
Ω aragonite	2.08 ± 0.10	1.38 ± 0.10
Temperature (°C)	22.79 ± 0.21	22.85 ± 0.21
Salinity (psu)	34.05 ± 0.2	35.05 ± 0.2
TA (μmol kg^−1^)	2431 ± 6	2420 ± 4
DIC (μmol kg^−1^)	2270 ± 13	2341 ± 11

TA, total alkalinity; DIC, dissolved inorganic carbon.

pH, Ω calcite and Ω aragonite values modelled from CO2SYS (Lewis & Wallace, 1998) with refitted constants (Mehrbach *et al*., 1973; Dickson & Millero, 1987)

### Nutrient analysis

Total phosphate and silicate measurements were performed by the Scottish Association of Marine Sciences using a Lachat 8500 Flow Inject Autoanalyser (Milwaukee, WI, USA) according to manufacturer's own methods (phosphate 31‐115‐01‐1‐I, silicate 31‐114‐27‐1‐A).

### Total alkalinity (TA; μmol kg^−1^SW) and total carbon dioxide (AKA DIC; TCO_2_; μmol kg^−1^SW)

Seawater was collected from each experimental tank with a clean 20 cm^3^ plastic pipette and placed in a clean glass pyrex bottle (WB40/80; SciLabware Ltd, Stoke‐on‐Trent, UK). Saturated mercuric chloride in deionized water was added to seawater samples to a concentration of 0.05% when bottles were sealed with a ground glass stopper coated with a thin layer of ultrahigh vacuum grease (Apiezon; SPI supplies, West Chester, PA, USA) to block air exchange. Samples were then stored at 4 °C until analysis. Both TA and DIC were measured by the Plymouth Marine Laboratory as previously described (Findlay *et al*., 2013). TA was measured in duplicate for each sample and the estimate of measurement error = 0.4%. Dissolved inorganic carbon was measured using a DIC analyser (Model AS‐C3; Apollo SciTech, Bogart, GA, USA). A measurement volume of 0.75 cm^3^ was used, with up to five measurements per sample. Values outside a 0.1% range were excluded from the final result. Duplicate measurements provided an estimate of measurement error = 0.2%. Chemistry parameters were evaluated using the CO2SYS spreadsheet (http://cdiac.ornl.gov/ftp/co2sys/CO2SYS_calc_XLS_v2.1/; Table 1).

### SEM studies

Plastic tiles were preserved in ethanol and used to investigate the detailed structure and appearance of the fouling spirorbids. Selected areas were cut from tiles and cleaned in an ultrasonic bath. Observations of gold‐coated samples were made using a Jeol 820 SEM at 20 kV (Welwyn Garden City, UK).

### Barcoding

The ascidians and sponge were identified to the genus level using 18s barcoding. Primers 18S‐SSUA NSF4 5′‐CTGGTTGATYCTGCCAGT‐3′, 18S‐SSUA NSR581: 5′‐ATTACCGCGGCTGCTGGC‐3′ in a standard PCR mix (Biotaq, Bioline, UK) with the following PCR conditions 94 °C 30 s, 40 cycles of 94 °C 30 s, 55 °C 30 s, 72 °C 1 min and a final step of 72 °C for 5 min.

## Results

In a pilot study of the effects of predicted change on the biofouling community at the CCMAR (Centre of Marine Science) experimental station (Ria Formosa lagoon, Portugal, 36°59′33″N 7°54′17″W), there was no temperature effect, but reduced pH affected both community structure and composition, when held at typical summer (24 °C) and autumn/spring (19 °C) values (online supporting material). In this investigation, we thus concentrated on acidification effects and conducted experiments at pH 7.9 (ambient) and pH 7.7 at a constant 23 °C and ambient salinity (Table 1). The pH reduction (0.2 pH units) was less than that predicted by the IPCC ‘business‐as‐usual’ scenario of a reduction of 0.3–0.4 pH units in oceanic surface waters by the year 2100, but will likely be achieved between 2055 and 2070.

**Figure 1:**
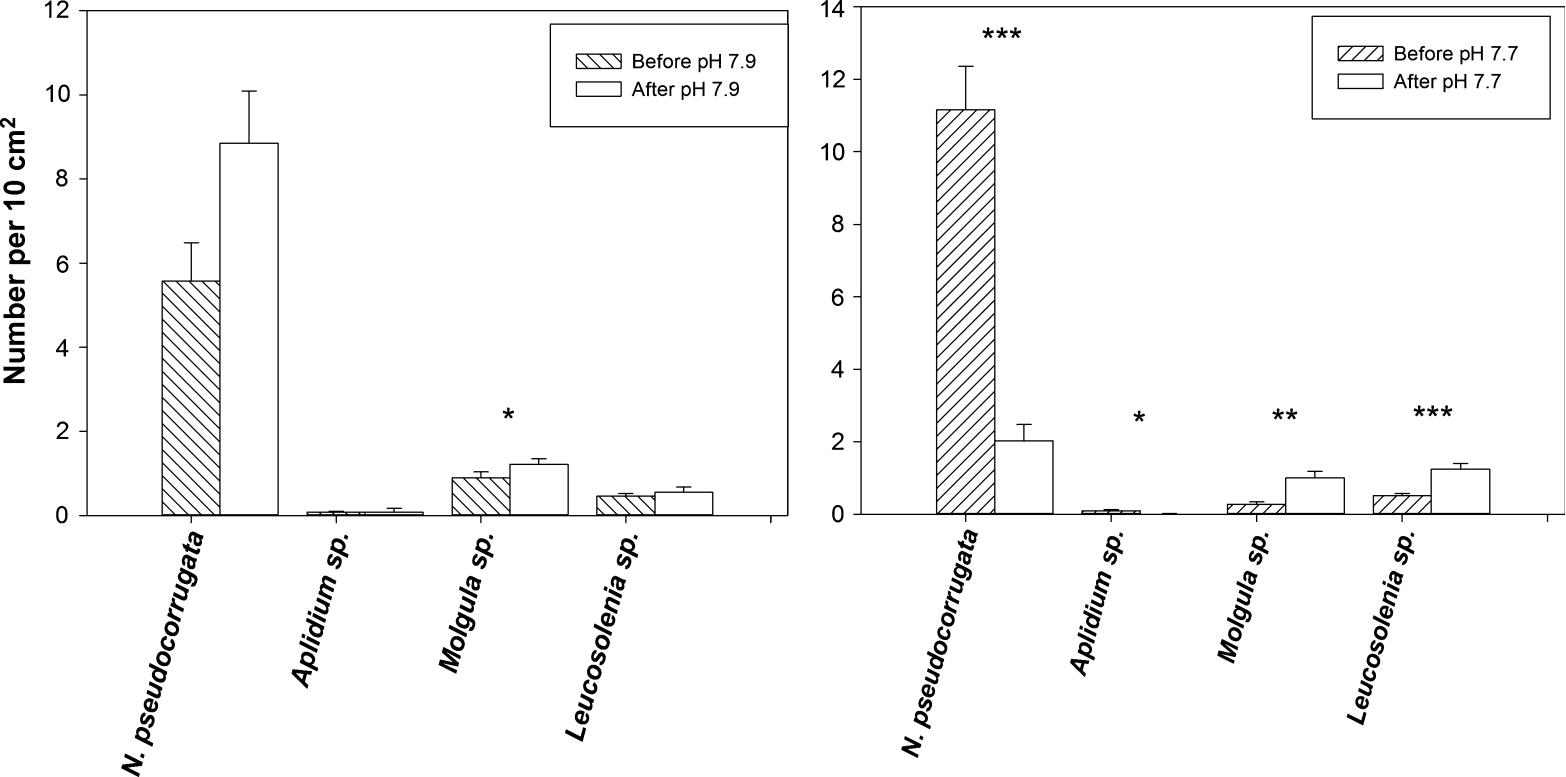
Numbers of the main components of the biofouling community on precolonized HDPE pipe before and after exposure to either pH 7.9 (control) of pH 7.7 (predicted year 2100 level). Values are mean per 10 cm^2^ ± SE; significant differences shown in figure as: *P < 0.01, **P < 0.001, ***P < 0.0001.

On the precolonized substrata, the initial community was dominated by the spirorbid polychaete *Neodexiospira pseudocorrugata* which accounted for 79.5–92.6% of the individuals present (Fig. 1). The other species present in high enough numbers to analyse effects of altered conditions were ascidians from species of the genus *Aplidium* (0.8–1.1%) and *Molgula* (2.3–12.8%), plus a sponge of the genus *Leucosolenia* (4.3–6.5%). In the controls (pH 7.9), there were no differences between the start and end of the trials in numbers of spirorbids (*H* = 3.27, 1 df, *n* = 60, *P* = 0.07), sponges (*H* = 3.35, 1 df, *n* = 90, *P* = 0.07) and the ascidian *Aplidium* sp. (*H* = 0.01, 1 df, *n* = 108, *P* = 0.92). There was a small (29%), but significant, increase in the ascidian *Molgula* sp. numbers at the end (*H* = 12.31, 1 df, *n* = 99, *P* < 0.01). Conversely, after 100 days exposure to pH 7.7, even though at this lower pH neither calcite nor aragonite was undersaturated, the community was changed markedly, with fewer spirorbids (47.4%), but more sponge colonies (*Leucosolenia* sp., 29%). In the ascidians, *Molgula* sp. were more common (23.4%) and *Aplidium* sp less at 0.2%. For all four taxa studied, new recruits were observed in all treatments. Spirorbid numbers decreased significantly from 11.1 ± 1.2 to 2.0 ± 1.2 individuals per 10 cm^2^ (*H* = 13.21, 1 df, *n* = 50, *P* < 0.0001); numbers of the ascidian (*Molgula* sp.) increased fourfold from the start to end of the trials (*H* = 9.73, 1 df, *n* = 90, *P* = 0.001); whilst the second, less abundant, ascidian (*Aplidium* sp.) decreased by an order of magnitude (*H* = 6.61, 1 df, *n* = 108, *P* = 0.01); and the sponge *Leucosolenia* sp. increased 2.5‐fold (*H* = 13.49, 1 df, *n* = 90, *P* < 0.0001).

The major change in community composition was due to the marked reductions in numbers of spirorbids at low pH, even though estimates were conservative. The counts quoted above only included living *N. pseudocorrugata*. Dead and destroyed individuals were visible from the remaining scar. The proportions of total numbers of spirorbids that were dead were not significantly different between treatments at the start of trials on precolonized pipe (pH 7.9, 19.7% (5.4% SE); pH 7.7, 12.6% (4.6% SE); *t* = 1.01, *P* = 0.332, 17 df). However, the proportion of dead individuals was significantly higher in the pH 7.7 treatment than in controls at the end of the experiment [pH 7.9, 23.3% (3.8% SE), pH 7.7, 72.8% (5.1% SE); *t* = 7.78, *P* < 0.0001, 17 df].

**Figure 2:**
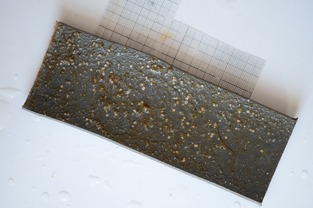
Section of HDPE pipe used in colonization trials.

**Figure 3:**
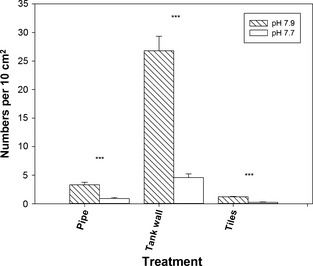
Colonization of new surfaces by the spirorbid *Neodexiospira pseudocorrugata* after 100 days exposure to either pH 7.9 (control) or pH 7.7. Values shown are means ± SE and presented as numbers per 10 cm^2^. All before and after differences were significant (Kruskal–Wallis tests, *H* > 18.6 in all cases) at *P* < 0.0001, indicated on figure by ***.

Biofouling communities colonize different materials with varying success. We therefore placed clean sections of high‐density polyethylene (HDPE) pipe that were open, having been cut lengthwise (Fig. 2), and clean limestone tiles into our system and also monitored colonization of cleaned PVC tank walls in the controls and pH 7.7 trials over the duration of the experiment. All three surfaces were open, and this avoided the possibility that metabolic effects due to enclosed areas could alter pH conditions. Densities of spirorbids differed markedly on the various surfaces at the end of the 100‐day trials (pH 8: *H* = 80.46, 2 df, *n* = 113, *P* < 0.0001; pH 7.7 *H* = 28.8, 2 df, *n* = 113, *P* < 0.0001) (Fig. 3). In pH 7.9, spirorbid colonization of tank walls was higher than tiles (*H* = 41.83, 1 df, *n* = 83, *P* < 0.0001) and HDPE pipe (*H* = 54.93, 1 df, *n* = 95, *P* < 0.0001) and pipe were higher than tiles (*H* = 15.71, 1 df, *n* = 48, *P* < 0.001). Reduced pH lowered spirorbid numbers on tank walls by nearly sixfold, on pipe by 3.5‐fold and on tiles by nearly fivefold, and all of these were significant (*H* = 74.33, 1 df, *n* = 130, *P* < 0.0001; *H* = 18.65, 1 df, *n* = 60, *P* < 0.0001; *H* = 25.13, 1 df, *n* = 36, *P* < 0.0001, respectively). Numbers of other taxa were too low to analyse after 100 days on new substrata.

Colonization of new surfaces by diatoms and filamentous algae was markedly different in the reduced pH trials compared to controls. It was not possible to quantify this effect from counts. Estimates were thus made visually from photographs of HDPE pipe surfaces and colonization levels classified into five categories from the lowest (1) to highest (5). Because this is a category analysis, nonparametric statistics were used to test for differences, and algal colonization in controls (pH 7.9; mean score = 4.4) was significantly higher than in low pH treatments (pH 7.7; mean score = 1.8; Mann–Whitney *W* = 45, *P* = 0.008, *n* = 11).

**Figure 4:**
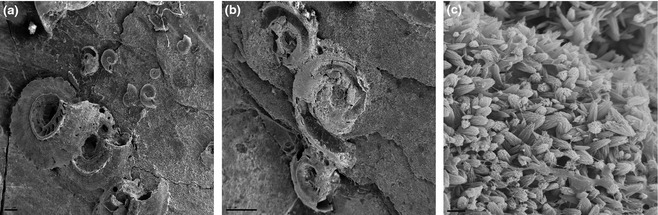
SEM images of typical spirorbid shells from specimens in the trials. (a) Spirorbid shells at end of trials held in ambient pH (7.9); (b) spirorbid shell remains at end of trials held at low pH (7.7); (c) high magnification view of spirorbid shell structure from low pH (7.7) treatment showing loose matrix of randomly aligned prisms. Scale bars a = 200 μm; b = 500 μm; c = 2 μm.

SEM analyses showed largely intact spirorbids with smooth outer surfaces from controls, but those at low pH were frequently ‘breached’ revealing internal structures (Fig. 4). XRD and SEM analyses confirmed the mineralogy as low magnesium calcite and an ultrastructure comprised predominantly of very small (<5 μm, Fig. 4) randomly aligned prismatic units, with little or no pitting or dissolution. There also appeared to be less binding matrix between prisms in spirorbid skeletons from the low pH treatments.

## Discussion

### Assemblage composition

Reductions in spirorbid numbers in low pH treatments were due to both ‘loss’ of adults and lack of recruitment. Recent studies have demonstrated that reduced pH does not affect metamorphosis in the tube worm *Hydroides elegans*, but it does affect larval and juvenile calcification (Lane *et al*., 2013), as well as weakening the adult calcareous tubes (Chan *et al*., 2012). Established spirorbids may be lost due to either detachment or degradation of tube material. The cement holding spirorbids to the substratum forms a very thin layer of mixed organics, high magnesium calcite and aragonite (Tanur *et al*., 2010) with little exposed surface for dissolution. Spirorbid loss here was thus most likely from degradation of tube material, which was confirmed by the SEM analyses showing largely intact individuals from controls, but in low pH, individuals were generally heavily damaged (Fig. 4). The analyses showing shell mineralogy as low magnesium calcite and shells composed of small irregularly orientated prisms are similar to shell structure reported previously for many spirorbids and other serpulids (Vinn *et al*., 2008). Despite the loose prismatic microstructure presenting a high surface area, our SEM investigation revealed no evidence of dissolution of the calcite prisms (Fig. 4). This was true in all pH treatments, even where losses were greatest in pH 7.7. Neither calcite nor aragonite, however, were below the saturation horizon in any treatment (Ω = 1, Table 1). The observations reported here thus indicate that degradation of the tubes was due to disintegration of the binding matrix rather than dissolution of crystals. The matrix is reportedly composed of an acid mucopolysaccharide (Vinn *et al*., 2008). Acid mucopolysaccharides are common constituents of connective tissue (Talwar & Srivastava, 2006). They are generally water soluble, and the solubility of many connective tissues and their products increases at lower pH (Tømmeraas & Melander, 2008). Some taxa may therefore be more susceptible to acidified conditions because their skeletons contain materials that dissolve more readily at reduced pH than CaCO_3_. Many other serpulids construct their tubes from high magnesium calcite or aragonite and, as they are more soluble polymorphs of CaCO_3_ than the low magnesium calcite in the *N. pseudocorrugata* studied here (Lowenstam, 1954; Vinn *et al*., 2008), these taxa may be more susceptible to dissolution (Smith *et al*., 2013) than those described here, although recent studies have reported serpulids from abyssal and hadal depths (Kupriyanova *et al*., 2014) where CaCO_3_ solubility is below the saturation horizon (Ω < 1), but pH is around 8. It is of note that the tubes of all serpulid worms lack a protective external organic sheet equivalent to molluscan periostracum (Tanur *et al*., 2010). Molluscs from high dissolution environments such as freshwater or the deep sea have particularly well‐developed periostraca (Vermeij, 1995).

### Differential sensitivity

The results here support previous findings that species with exposed CaCO_3_ skeletons are impacted heavily by acidified conditions (Orr *et al*., 2005). This appears to be especially so for encrusting biofouling communities, where groups like spirorbid worms exhibit relatively ephemeral, r‐selected, life‐history strategies of colonizing, growing rapidly and achieving reproductive size early (Bowden *et al*., 2006). Our data indicate such species are amongst the most vulnerable to reduced pH. Their unprotected exoskeletons constructed of fine crystals embedded in material that appears more soluble at low pH make them more vulnerable. This view is further supported by the poor colonization and survival of spirorbids in CO_2_ vent areas in the Mediterranean (Cigliano *et al*., 2012).

Soft‐bodied marine species, or those with protective organic coatings, or living tissue covering their skeletons seemingly have few problems maintaining their integrity at lowered pH. All marine organisms face the challenge of increased costs of pumping carbonate ions to maintain cellular homeostasis, and those which secrete calcium carbonate (even when protected by organic sheaths) must create the correct saturation conditions at the site of biomineralization. Species with organic coverings of their skeletons may be more resistant to low pH, including the Antarctic sea urchin *Sterechinus neumayeri*, which has been held in pH 7.7 and 7.4 for more than 2 years without mortality, and individuals fed well and produced viable gametes after this time (Suckling *et al*., 2014).

### Mechanisms

The mechanisms responsible for increasing numbers in low pH as seen here for the ascidian *Molgula* sp. and the sponge *Leucosolenia* sp. include reduced competition for space (Todd, 1998; Bowden *et al*., 2006). *Leucosolenia* produces calcite spicules that are protected in organic sheaths (Jones, 1955). Our data, however, do not support the prediction that calcareous sponges will necessarily be losers in the event of increased OA, although in naturally high CO_2_ sites, siliceous sponges are more successful (Goodwin *et al*., 2014). Previous work has indicated that settlement and recruitment is neither facilitated, nor inhibited by previous colonists in biofouling communities (Watson & Barnes, 2004; Bowden *et al*., 2006), hence either low pH directly improved conditions for recruitment and growth of our ascidians, or numbers increased because of reduced competition.

### Broader implications

Impacts on biofouling communities from a marked decrease in calcified groups and an increase in soft‐bodied forms would include a slower build‐up of biomass, because the latter are not amongst the earliest colonizers. Such communities will have reduced 3‐D complexity, likely providing less habitat for secondary colonization. Requirements for antifouling would change. There would be less emphasis on removal of species with hard encrusting skeletons and more on deterring recruitment of subsequent groups, which could reduce costs, especially in aquaculture industries, where detrimental biofouling impacts can develop rapidly. The removal of encrusting groups with unprotected carbonate exoskeletons and the increase of species such as the ascidians here have marked implications for the biology and conservation of encrusting communities worldwide.

## Supporting information


**Appendix S1**. Supplementary Material.
**Table S1.** Data of physical parameters measured in the experimental tanks.
**Figure S1.** Numbers of individuals of the main biofouling species on oyster shells in the 4 treatments used.
**Figure S2.** Images of oyster shells with biofouling organisms at the end of trials to show spirorbid numbers.
