# Supplementary material for: Acidification effects on biofouling communities: winners and losers
Source: Glob Chang Biol. 2015 Jan 28;21(5):1907–13. doi: 10.1111/gcb.12841 (PMC5006883; doi:10.1111/gcb.12841)
Supplement: Supplementary file 1 — Appendix S1. Supplementary Material. Table S1. Data of physical parameters measured in the experimental tanks. Figure S1. Numbers of individuals of the main biofouling species on oyster shells in the 4 treatments used. Figure S2. Images of oyster shells with biofouling organisms at the end of trials to show spirorbid numbers. [file GCB-21-1907-s001.doc]

**Online supporting material:**

*Pilot Study:*

Experiments to assess the impact of temperature (18^o^C and 23^o^C) and pH (7.9 and 7.5) on biofouling communities on shells of oysters (*Crassostrea gigas*) took place in the experimental station of CCMAR (Centre of Marine Science) in the Ria Formosa lagoon (36°59'33"N   7°54'17"W, Portugal).

**Methods**

Animals from the same cohort, of oysters were obtained from the Arcachon Bay hatchery (SW France) and cultivated for one year in the Ria Formosa lagoon (Fuzeta), prior to the start of these experiments. They were placed in a flow-through holding system, which consisted of 4 tanks: 23°C normal pH, 23°C low pH, 18°C low pH and 18°C normal pH and was supplied with sea water from the Ria Formosa lagoon, which had been pre-treated through a sand-filter. The system comprised two separate circuits for the 18ºC and 23ºC treatments. Each circuit had a 250 L header tank and two experimental 50L tanks, which housed the oysters, one for each pH treatment. The header tank received the water from the main aquarium supply additionally filtered through a 20micron inline filter (polyethylene nylon wool), this, then delivered water to the experimental tanks by gravity. The water was aerated to assure adequate oxygenation and stable pH, and the temperature was adjusted. Each experimental tank with the oysters was continuously aerated and received 150ml/min of saltwater from the header tank, the excess water overflowed so that the volume in each tank was totally exchanged 3-4 times per day. The experimental tanks were illuminated with day light fluorescent lamps with a photo period 12h/12H (light/dark). Water temperature in both header tanks was controlled using a refrigerator and a heater equipped with a digital thermostat (+-1ºC). The experimental tanks were installed inside polystyrene boxes to reduce the temperature fluctuations. pH was set in the low pH tanks to 7.5. To reduce the sea water pH, aeration was enriched with CO_2_ and the CO_2_ injection was controlled by an Aqua Medic - pH computer-Set and a solenoid valve. The pH level in the pH 7.9 experimental tanks was obtained by continuously aeration of the water tank. The temperature in the experimental tanks and header tanks was recorded every 30 min using a Maxim –Dallas data logger (DS1920L) with 0.5ºC precision. The output from both CO2 and temperature monitoring was used in real time to modify pH and/or temperature. The salinity was measured with a VWR EC 300 – conductivity meter and the pH was measured with a OxyGuard Handy pH meter. The oysters were fed with a mixture of the microalgae *Isochrysis galbana* (clone T-ISO) and *Tetraselmis Suecica* in a proportion of 1:1 in terms of size, supplied in continuous flow to each tank by a peristaltic pump (ISMATEC MPC Process) at a standard concentration of 18,000 cells/ml^-1^ per experiment. The concentration of chlorophyll-a (Chl-a) in each tank was measured *in-vivo*, using a portable fluorometer (10 AU-Turner Designs. This food was available to the biofouling organisms on the shells. Oysters were photographed to allow quantification of the biofouling community using NIKON D80 with a NIKON DX SWM ED IF Aspherical AF-S NIKKOR 18-70 mm 1:35.5-4.5G ED lens before they were introduced into the experimental circuit. Shells were numbered with an indelible pen to facilitate matching of individual oysters during the experiment. At the end of the experiment photographs were taken of the upper and lower surface of all oysters.

**Results**

In this pilot study live oysters (25-30) with associated community were macro photographed and placed in a latin square designed experiment with temperatures of 24°C (summer) or 19°C (winter) and pH of either 7.9 (ambient) or 7.5 with ambient oxygen and salinity values for inshore sites near Faro (Centre of Marine Science, Ria Formosa lagoon; 36°59'33"N   7°54'17"W), Portugal where the experiments were conducted (Table 1). They were held in each treatment for 100 days. Numbers of epibionts were counted from photographs, but identified live. The initial community was dominated by spirorbid polychaetes (very abundant *N. pseudocorrugata* and infrequent *S. pseudomilitaris*), which accounted for 1875 of the 1926 visible epibionts on the oysters (Fig S1). The rest of the community comprised the barnacle *Elminius modestus* Darwin, 1854 (41 individuals), 7 unidentified solitary ascidians and 1 unidentified bryozoan colony. At the end of the trials spirorbids had increased significantly in numbers in both summer (x28.4 increase) and winter (x10.4 increase) temperature treatments in normal pH, but had decreased markedly at low pH in summer (x 0.31 of start number) and winter (x 0.39 of start number) temperatures. A GLM analysis on final numbers showed that changes due to temperature were not significant (F_1,200_=0.12, P=0.72, 1, 200 df), whereas pH effects were significant (F_1,200_=196.9, P<0.001), and there was no interaction (F_1,200_=0.49, P=0.49). Thus high temperature had no detectable effect on spirorbid polychaetes, but low pH dramatically reduced numbers. Furthermore the counts made were of remaining tubes, and limited more detailed observations suggested that most, if not all spirorbid tubes did not contain living worms at the end of low pH treatments. Our data should thus be viewed as the maximum number of individuals surviving in low pH. Most tubes contained live individuals in normal pH trials. A noticeable further difference was that at the end of normal pH treatments large numbers of very small, newly colonised individuals were present, but these were absent in low pH treatments. Similarly to the spirorbids a GLM analysis of final barnacle numbers, although much lower than spirorbids, showed they were not affected by temperature (F_1, 200_=1.39, P=0.24), but were strongly reduced in low pH (F_1, 200_=11.36, P<0.001) and there was no interaction effect (F_1, 200_=1.39, P=0.24). A difference from the spirorbids, however, was that barnacle skeletons were still present, but empty. Further to these observations at the start of trials experimental specimens were placed in clean plastic mesh baskets. At the end of trials the control mesh baskets were colonised with spirorbid polychaetes at both temperatures, but these were absent in low pH trials, (Fig S2.)

In contrast to the dramatic negative impacts on spirorbids and barnacles, ascidian numbers increased in all treatments. Again there was no temperature effect on final numbers (GLM, F_1,200_=0.16, P=0.69), but numbers were significantly higher in low pH (GLM, F_1,200_=8.1, P=0.005), and there was no significant interaction effect (GLM, F_1, 200_=0.02, P=0.89). Acidified conditions thus improved survival and recruitment of ascidians irrespective of temperature. In terms of overall numbers ascidians increased from less than 0.1% of the total to 19.4% in low pH treatments, but remained at very low levels in normal pH. Other taxa present in the study (serpulid worms, flatworms and the bivalve *Mytilus edulis*), either at the start or end, were present in numbers that were too small to be able to identify temperature or pH effects.

**Table S1:** Data of physical parameters measured in the experimental tanks. Results are shown ± SE. Temperature data was measured directly in the experimental tanks, the data loggers were used in the header tanks only and for the 18°C experiment, the mean water temperature was 18.40°C ± 0.02 SE and for the 23°C experiment: 23.20°C ±0.2 SE.

|  | **Tank 1** | **Tank 2** | **Tank 3** | **Tank4** |
| --- | --- | --- | --- | --- |
|  | **24°C, normal pH** | **24°C, low pH** | **19°C, low pH** | **19°C, normal pH** |
| **Temperature** | 24.3 ± 0.7 | 24.2 ± 0.3 | 18.8 ± 0.1 | 19.4 ± 0.5 |
| **pH** | 7.89 ± 0.01 | 7.45 ± 0.02 | 7.46 ± 0.02 | 7.84 ± 0.02 |
| **Salinity (%º)** | 35.9 ± 0.3 | 35.9 ± 0.3 | 36.2 ± 0.1 | 35.5 ± 0.5 |
| **Oxygen (%)** | 88.2 ± 2.8 | 88.3 ± 2.6 | 82.9 ± 4.9 | 87.9 ± 3.2 |
| **Oxygen (mg/L)** | 6.25 ± 0.37 | 6.54 ± 0.07 | 7.15 ± 0.05 | 7.17 ± 0.05 |

Figure Legends

Fig S1. Numbers of individuals of the main biofouling species on oyster shells in the 4 treatments used. Open bars are numbers at the start of trials, hatched bars are numbers at the end of trials.

Fig S2. Images of oyster shells with biofouling organisms at the end of trials to show spirorbid numbers. A. Summer temperature, ambient pH (7.9); B. Summer temperature, low pH (7.6); C. Winter temperature, low pH (7.6); D Winter temperature ambient pH (7.9). Note also the colonisation of the plastic mesh supporting oysters in ambient pH trials, but their absence in low pH trials.

Figure S1


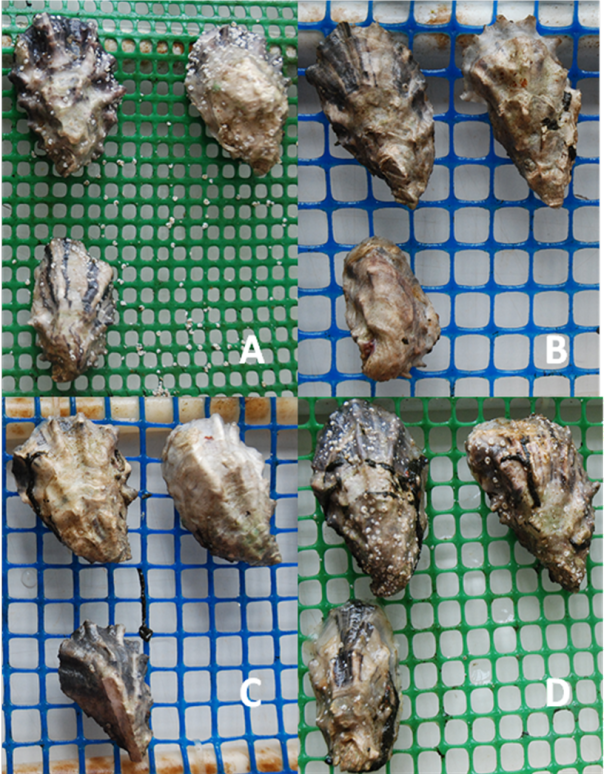


Figure S2
